# Evaluation of sampling effort required to assess pollen species richness on pollinators using rarefaction

**DOI:** 10.1002/aps3.11411

**Published:** 2021-02-27

**Authors:** Aoi Nikkeshi, Masayoshi K. Hiraiwa, Atushi Ushimaru, Kazuhiko Hoshizaki, Akifumi Makita, Inoue Mizuki

**Affiliations:** ^1^ Division of Biodiversity Institute for Agro‐environmental Science NARO 3‐1‐3, Kannondai Tsukuba Ibaraki 305‐8604 Japan; ^2^ Department of Biological Environment, Faculty of Bioresource Sciences Akita Prefectural University Shimoshinjo‐Nakano Akita 010‐0195 Japan; ^3^ Graduate School of Human Development and Environment Kobe University Kobe Hyogo 657‐8501 Japan; ^4^ Department of Biosciences College of Humanities and Sciences Nihon University 3‐25‐40 Sakurajousui Setagaya‐ku Tokyo 156‐8550 Japan

**Keywords:** deposited pollen, microscopic observation, network links, pollen species richness, pollination network, rarefaction

## Abstract

**Premise:**

Understanding the flower visitation history of individual pollinators is key in the study of pollination networks, but direct tracking is labor intensive and, more important, does not capture information about the previous interactions of an individual. Therefore, a protocol to detect most of the pollen species on the body surfaces of an individual pollinator could elucidate its flower visitation history.

**Methods and Results:**

Under a microscope, we observed 6.0‐µL droplets from a sample solution (1.0 or 3.0 mL) containing pollen grains collected from individuals of six major pollinator functional groups. To clarify how many droplets need to be observed to detect all pollen species within the solution, we examined up to 10 droplets collected from each individual insect. Sample‐based rarefaction curve analyses of the data showed that we could detect ~90% of the pollen species and the plant–pollinator links in the networks by observing six droplets.

**Conclusions:**

The rarefaction curve analysis for pollen‐on‐pollinator studies is a useful preliminary step for minimizing the time and labor required while maximizing the data on the flower visitation history of each individual pollinator and revealing any hidden flower–pollinator interactions.

Revealing the flower visitation history of pollinator individuals provides basic and useful information for studying pollination networks (Alarcón, 2010; Orford et al., 2015) and the spatiotemporal flexibility of flower preference and constancy (e.g., Heinrich, 1975; Martin and Farina, 2016). Flower visitation history has traditionally been examined by the direct tracking of pollinators in the field (Osborne et al., 1999). These methods are labor intensive when tracking multiple visitations by each of many individual insects and when examining long‐distance pollinators, such as butterflies and moths, and do not capture information about the previous interactions of an individual, which is the more serious issue. The examination of pollen grains on the bodies of multiple pollinators has instead become an essential method that complements direct pollinator observation (Alarcón, 2010; Tur et al., 2015). The examination of pollen grains deposited on flower visitors is important not only in examining whether the insects effectively pollinate a given plant species, but also in revealing reproductive interference among plant species (Morales and Traveset, 2008). In addition, pollen‐on‐pollinator data are used to study the robustness of pollination networks (Memmott et al., 2004), the effects of the loss of a single pollinator on pollination function (Brosi and Briggs, 2013), and the temporal food resource changes of pollinators during their lifespan (Di Pasquale et al., 2013).

Generally, pollen‐on‐pollinator studies are based on the microscopic observation of sample solutions containing pollen grains washed or removed from pollinator body surfaces using, for example, a water solution (Parker, 1981), ethanol solution (Goldblatt et al., 1989), sucrose solution (Nikkeshi et al., 2016, 2019), or fuchsin jelly (Kearns and Inouye, 1993). Recently, DNA barcoding has been used to analyze the pollen species on pollinators (Bell et al., 2016), but it is expensive when applied to many samples and inevitably requires DNA reference databases, which are often not well established for plant species in the target areas. Thus, microscopic observation is still useful for many pollination ecologists. All pollen grains within a sample solution should ideally be examined and identified; however, this is time and labor intensive when there are many pollen grains in each sample and the number of samples is large. Therefore, the number of pollen grains in a sample is usually determined by counting the grains in a fixed volume of a few droplets from a given sample solution under a microscope, then the total number of pollen grains in the solution is estimated by scaling up the count. However, observing only a limited number of droplets might pose a risk of missing some pollen species in the solution, and thus the appropriate number of droplet observations per solution should be determined to develop a protocol for pollen‐on‐pollinator studies. This number may differ among pollinator functional groups that carry different amounts of pollen grains owing to differences in body surface area, the presence/absence of hair, and behaviors on flowers, although no studies have examined this issue.

Here, we determined the appropriate number of droplet observations for six major pollinator functional groups (large bees, medium bees, syrphid flies, other dipterans, lepidopterans, and beetles) by examining 91 pollinator individuals previously used to establish a method of pollen collection from their body surfaces in sucrose solution (Nikkeshi et al., 2016). In that previous study, we found that the method collected a mean of 96.2% (median of 99.2%) of the pollen grains on each individual into the solution without pollen damage or decolorization, each of which decreases the accuracy of species identification (Table 1). Using the same sample solutions, we examined how many droplet observations were required to detect all the pollen species on each pollinator individual. We discuss the appropriate number of droplet observations for each pollinator group based on our results.

**TABLE 1:** The average number of pollen grains deposited on pollinator individuals and recovery percentage by sucrose solution of each functional group by microscopic observation (mean ± SE).

Pollen grain recovery^a^	Pollinator group					
	Large bees (*N* = 27)	Medium bees (*N* = 19)	Syrphid flies (*N* = 6)	Other dipterans (*N* = 20)	Lepidopterans (*N* = 10)	Beetles (*N* = 9)
No. ± SE	1194.3 ± 237.6	1529.3 ± 678.4	28.0 ± 12.8	102.5 ± 67.4	3.4 ± 3.7	1246.7 ± 678.4
% ± SE	92.9 ± 2.33	93.2 ± 0.76	96.7 ± 3.83	97.8 ± 1.45	100.0 ± 0.00	98.5 ± 0.76

Note

*N* = number of individuals sampled.

^a^ The counted number of pollen grains and the recovery percentage on the pollinator body in the previous study (Nikkeshi et al., 2016).

## METHODS

### Study site and pollinator collection

We examined pollinators at Koizumigata Park (63.7 ha in area), Akita Prefecture, northern Japan (39°48′45″N, 140°04′20″E). We set and surveyed a line plot (ca. 2 km in length, 4 m in width) over seven days from late September to early October 2009 (24, 26, 28, and 29 September; 1, 4, and 12 October). The vegetation in the park was dominated by secondary forests, and semi‐natural grasslands were maintained by mowing in open spaces and along forest edges and roads. We recorded the species names of all plants that flowered within the line plot every 2 h from 06:00 to 18:00 for six days and from 18:00 to 06:00 over a single night starting on 28 September, then collected the pollinators in a nylon net and recorded their host flowers. We stored each individual separately in a 1.5‐mL or 15‐mL tube (depending on body size) and stored them at −30°C until required for pollen removal.

The collected pollinators were identified by their morphology and classified into six taxonomic functional groups (Nikkeshi et al., 2016): large bees (nine *Bombus diversus diversus*, nine *Xylocopa appendiculata circumvolans*, and nine *Megachile sculpturalis* individuals), medium bees (eight *Apis* spp., three *Megachile* spp., and eight *Tetraloniella mitsukurii* individuals), syrphid flies (eight *Episyrphus balteatus* individuals), other dipterans (eight *Lucilia illustris*, six other *Lucilia* spp., three Anthomyiidae, and three *Tipula* spp. individuals), lepidopterans (12 *Parnara guttata* and eight Pyraustinae sp. individuals), and beetles (nine *Oxycetonia jucunda* individuals). We collected plant and pollen specimens for all plants with flowers at the study site during the study period. A few flower buds of each plant species were collected and stored together in a 1.5‐mL tube. The bud samples were dried in silica gel at room temperature for the collection of pollen specimens.

### Pollen observation by microscope

We used 1.0 or 3.0 mL of 0.4 M sucrose solution to remove the pollen grains from pollinator bodies to overcome problems in existing pollen collection methods, such as the incomplete pollen removal in fuchsin gel (Kearns and Inouye, 1993) and pollen destruction and decolorization in water and ethanol solutions (Nikkeshi et al., 2016). Sucrose solution is also suitable for pollen observation because its high viscosity keeps pollen grains in suspension.

First, we created the 0.4 M sucrose solution by dissolving 136.8 g sucrose in 1000 mL of distilled water. Next, we added the solution into each sample tube and vortexed it well (30–60 s) to wash as many pollen grains as possible off the pollinator’s body surface. For large bees, some medium bees, beetles, and lepidopterans, 3.0 mL (15‐mL tube) of sucrose solution was used, and 1.0 mL (1.5‐mL tube) was used for the rest of the pollinators (Nikkeshi et al., 2016, 2019). By counting the number of pollen grains remaining on each pollinator after washing, we confirmed that the method collected more than 96.5% of pollen grains on each individual for all pollinator groups other than bees, which had a removal rate of ca. 93% (Nikkeshi et al., 2016; Table 1).

For each droplet observation trial, each tube was re‐vortexed to distribute the pollen grains uniformly in the solution. Next, a 6.0‐µL droplet of the solution was placed on a glass slide (76 × 26 mm) and covered with a cover slip (18 × 18 mm). Under a light microscope (Nikon Eclipse E600; Nikon, Tokyo, Japan; mostly 100× and 400× magnifications for small pollen grains <20 μm), we identified and recorded the species of all pollen grains for each individual droplet. Observation trials were repeated until no new pollen species were found in three consecutive trials for each solution, or were performed four times if no pollen grains were found in any droplets from the same pollinator. We identified pollen grains at the species level, where possible, on the basis of several pollen traits (size, morphology, color, pore size and shape, surface pattern, texture of protoplasm, and other characteristics), referring to the pollen specimens we collected at the study site. To make a reference specimen for each species, the pollen grains from each bud sample were mounted in 6.0 µL of sucrose solution on a glass slide with a cover slip. We also referred to the pollen sizes and morphologies reported in previous studies (Ikuse, 1956; Huang, 1972; which both contain electron micrographs). When we could not identify the pollen to the species level, we identified them to a genus or family, with a few exceptions which were grouped as “unknown species” (Appendix 1).

### Statistical analysis

For each pollinator individual, we created a one‐droplet pollen‐species table in which presence/absence (coded as 1/0) data were input for each pair of trial number (1–10) in rows and pollen species in columns. We estimated the number of pollen species on each pollinator individual from a sample (droplet)‐based rarefaction curve drawn from each one‐droplet pollen species table using the iNEXT function of the iNEXT package in R version 3.6.1 software (Chao et al., 2014; R Core Team, 2019; Hsieh et al., 2020). Despite likely representing multiple species, all unknown pollen was grouped as a single unknown species. The maximum number of droplet observation trials was 10, because no more new pollen species were detected on any individual after the 10th trial. We then calculated the average percentage of pollen species detected at each trial number for each functional group by assuming the maximum to have been reached at the 10th trial.

We also conducted a rarefaction curve analysis of the number of links in the plant–pollinator network. Although this required data from 10 droplets from each pollinator individual, fewer than 10 droplets were collected from most individuals (Appendix 2); therefore, we added randomly selected droplet data from the original data to make up the numbers. So, for example, if we had five droplets from a given individual, we added another five data points by random selection from the original five‐droplet data. We randomly assigned an identity number (1–10) to each of the 10 droplets for each pollinator individual. We next created 10 pollen species–pollinator networks using data from the same numbered droplets from all individuals; we identified each network with the droplet identity number. Then we created a table in which the network identity numbers (1–10) were set in rows and links between all observed pollen species–pollinator pairs were set in columns; the presence/absence (1/0) of each link was the input. From the data in this table, we estimated and drew a sample (droplet)‐based rarefaction curve of the link number within the study network using the iNEXT function.

## RESULTS

We observed 66 flowering species and collected 103 individuals of 13 pollinator taxa at the study site (Appendix 1): 27 large bees, 19 medium bees, eight syrphid flies, 20 other dipterans, 20 lepidopterans, and nine beetles. The pollinators were captured from 14 flowering species (Appendix 1). We excluded data from 10 lepidopterans and two syrphid flies that carried no pollen grains from the following analyses. The pollen grains collected from the 91 remaining pollinators were assigned to 22 pollen species: 16 identified and six unknown species (Appendix 1). We also found pollen of seven plant species (*Mazus pumilus* (Burm. f.) Steenis and six unknown taxa) whose flowers were not observed in the field. In total, we examined 73 species (66 flowering, one identified species, and six unknown taxa) in the study (Appendix 1). Seven pollinator individuals carried unknown pollen grains. The mean pollen species richness across the six functional groups was 1.8 ± 1.4 (mean ± SD) per individual (large bees, 2.96 ± 1.72; medium bees, 1.58 ± 0.69; syrphid flies, 1.33 ± 0.82; other dipterans, 1.50 ± 0.69; lepidopterans, 1.70 ± 1.34; beetles, 2.33 ± 1.22). The mean numbers of observation trials required per individual were higher in the large pollinator groups (large bees and beetles) than in the other groups (Appendix 2), with ≤6 required for most of the individuals (71 individuals; Appendix 2).

**TABLE 2:** Predicted numbers of pollen species (mean ± SD) detected in each number of observation trials for each pollinator functional group, estimated using a rarefaction curve analysis.

	Pollinator group					
No. of observation trials	Large bees (*N* = 27)	Medium bees (*N* = 19)	Syrphid flies (*N* = 6)	Other dipterans (*N* = 20)	Lepidopterans (*N* = 10)	Beetles (*N* = 9)
1	1.62 ± 0.70	0.96 ± 0.22	0.69 ± 0.21	0.84 ± 0.40	0.65 ± 0.52	0.94 ± 0.57
2	2.15 ± 0.96	1.24 ± 0.32	0.98 ± 0.30	1.11 ± 0.49	1.04 ± 0.78	1.34 ± 0.77
3	2.37 ± 1.13	1.32 ± 0.40	1.11 ± 0.40	1.21 ± 0.50	1.28 ± 0.94	1.56 ± 0.90
4	2.52 ± 1.26	1.40 ± 0.47	1.19 ± 0.49	1.29 ± 0.53	1.43 ± 1.03	1.74 ± 1.00
5	2.65 ± 1.36	1.46 ± 0.54	1.24 ± 0.56	1.37 ± 0.57	1.53 ± 1.10	1.90 ± 1.08
6	2.76 ± 1.45	1.51 ± 0.59	1.28 ± 0.62	1.42 ± 0.59	1.62 ± 1.17	2.03 ± 1.15
7	2.85 ± 1.53	1.54 ± 0.62	1.30 ± 0.66	1.46 ± 0.63	1.68 ± 1.20	2.14 ± 1.19
8	2.92 ± 1.60	1.56 ± 0.65	1.31 ± 0.70	1.50 ± 0.67	1.71 ± 1.25	2.20 ± 1.23
9	2.98 ± 1.67	1.57 ± 0.67	1.33 ± 0.75	1.52 ± 0.72	1.74 ± 1.28	2.24 ± 1.27
10	3.02 ± 1.74	1.59 ± 0.69	1.33 ± 0.75	1.54 ± 0.76	1.76 ± 1.31	2.27 ± 1.30

Note

*N* = number of individuals sampled.

**FIGURE 1:**
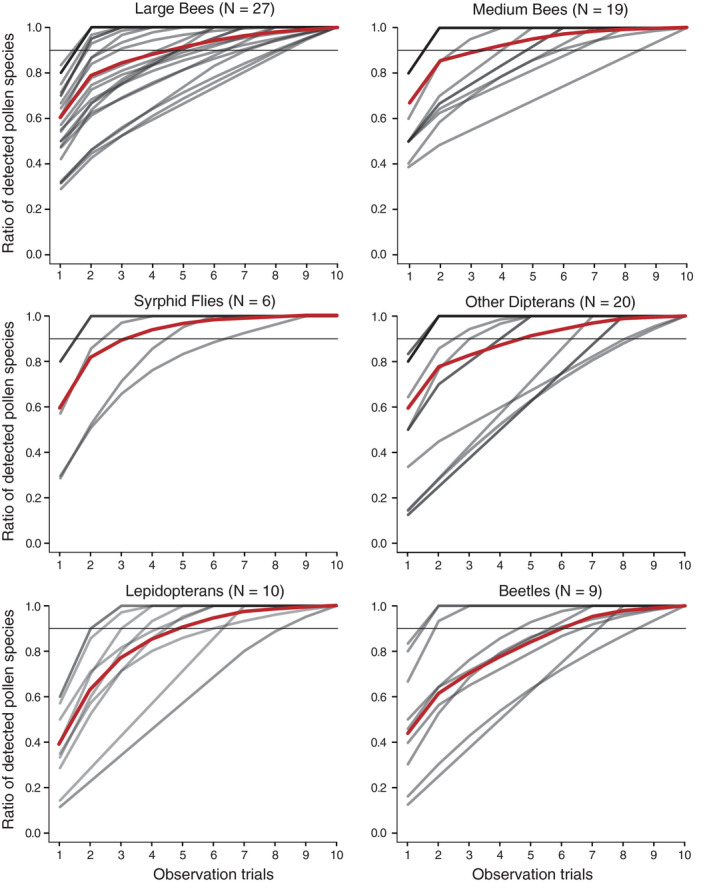
Rarefaction curves of detected pollen species for each individual (darker gray lines indicate overlap) and for the average of each pollinator group (red line). The sample size is given in parentheses. The horizontal gray line indicates 90% of all pollen species.

Among the six functional groups, the minimum estimated pollen species richness was 1.33 ± 0.75 on syrphid flies and the maximum was 3.02 ± 1.74 on large bees (Table 2). Rarefaction curves revealed mean rates of detection of pollen species of 84.1% ± 19.9% (mean ± SD) in three trials, 94.7% ± 2.6% in six trials, and 98.6% ± 0.6% in eight trials across the six functional groups. More than 95% of the pollen species on most individuals were found within observations of eight droplets (Fig. 1).

**FIGURE 2:**
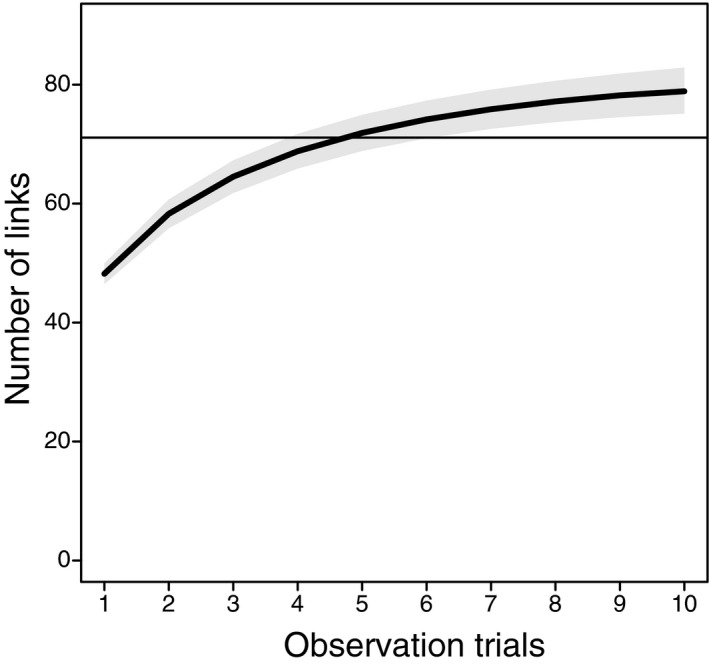
Rarefaction analysis for the number of plant–pollinator links in the study network. The black line and gray shading indicate the mean and the 95% confidence interval, respectively. The horizontal gray line indicates 90% of links, assuming the maximum links were detected at the 10th trial.

The rarefaction curve for the network link number revealed 79 links in 10 droplets. The observation of six droplets enabled the detection of 74.2 links (93.9% of the links in 10 droplets), and the number of newly detected links increased slightly with the number of observation trials (Fig. 2).

## CONCLUSIONS

Applying rarefaction analyses to the droplet observation data set, we showed that the number of pollen species detected on each pollinator and the number of links in the study network increased as the number of droplets examined from each individual increased. Six droplets was found to be an appropriate number of observations to reveal ca. 90% of the flower visitation histories of all pollinator groups—from large bees, which carried numerous diverse pollen grains, to syrphid flies and lepidopterans, which carried few pollen grains—and to detect ca. 98% of links in the study network (Figs. 1, 2). The optimal number of droplets to detect all pollen species on each pollinator individual was 10, whereas six observations missed ~10% of pollen species and three observations missed ~15% on average. However, even with three or six observations, very few pollen species would be undetected, because the pollen species richness on a single pollinator was <10 in most cases (A. Nikkeshi, personal observation). Although the observation of 3–6 droplets should be appropriate when assessing pollen species on each individual and the links in the system, care should be taken when applying our method to pollinators that carry more pollen grains and to ecosystems with a wider range of species. We observed 66 flowering species and diverse pollinator groups during the relatively short period (20 days) at the study site. Plant richness was higher in our study site and period than that in some seasons in species‐rich temperate forests and meadows (e.g., Inoue et al., 1990; Kato et al., 1990; Yamazaki and Kato, 2003). Six observation trials can therefore be appropriate for some temperate or more species‐poor ecosystems, although when plant–pollinator interactions are examined for longer periods, flowering phenology surveys and reference‐pollen collections should be repeated for pollen identification. On the other hand, more droplet observation trials per individual may be required for more plant species‐rich ecosystems, such as tropical rainforests where a large number of species flower synchronously within a short period (Sakai et al., 1999). Moreover, for ecosystems dominated by large pollinators, a larger number of trials might be needed. In any case, we recommend first clarifying how many observation trials are necessary until no new pollen species are found in each sample solution, and then deciding on the number of droplet observations appropriate for the study purpose using a rarefaction analysis. Finally, it must be noted that our capture method is destructive and may not be suitable for studying endangered pollinator species.

## AUTHOR CONTRIBUTIONS

A.N., K.H., A.M., and I.M. designed the study. A.N. collected the data. A.N., M.K.H., and A.U. analyzed and interpreted the data. A.N., M.K.H., and A.U. drafted the manuscript. All authors revised the manuscript and approved the final version.

## APPENDIX 1 List of 73 flowering species in the study site and the detected pollen species on pollinators.

**Table:**

Family	Species		Fl^a^	No. of insect individuals^b^						Type^c^	Size 1 (µm)^d^	Size 2 (µm)^e^
				LB	MB	SF	OD	L	B			
Anacardiaceae	*Rhus javanica* var. *chinensis* (Mill.) T. Yamaz.		○			1(1)	3(3)			6Bb	20–23	28–33
Apiaceae	*Angelica pubescens* Maxim.		○	(20)	(4)	(2)	(4)	(3)	(1)	6Bb	27–28	17.5
	Apiaceae sp.							(1)		6Bb	n.d.	n.d.
	*Oenanthe javanica* (Blume) DC.		○							6Bb	25.5–27	16
Araliaceae	*Aralia cordata* Thunb.		○							6Bb	24–26	24.5–27.5
Asparagaceae	*Hosta sieboldii* var. *rectifolia* (Nakai) H. Hara		○							2Aa	66–70	82–95
Asteraceae	*Aster iinumae* Kitam.		○							6Bb	23–25.5	24–26
	*Aster scaber* Thunb.		○	(4)	(1)			(1)	(5)	6Bb	27–28	28–29.5
	Asteraceae sp.				(1)					6Bb	n.d.	n.d.
	*Bidens frondosa* L.		○							6Bb‐c	32	33
	*Carpesium divaricatum* Siebold & Zucc. var. *divaricatum*		○							6Bb	23.5–25	24–25.5
	*Carpesium divaricatum* var. *matsuei* (Tatewaki & Kitam.) Kitam.		○							6Bb	24	25
	*Carpesium glossophyllum* Maxim.		○							6Bb	23.5	24
	*Cirsium japonicum* Fisch. ex DC.		○	(8)		2(2)	3(3)	4(5)	9(9)	6Bb	43–45.5	24.5–48
	*Coreopsis tinctoria* Nutt.		○							6Bb	25	25.5
	*Crassocephalum crepidioides* (Benth.) S. Moore		○							6Bb	31–32	31–34
	*Crepidiastrum denticulatum* (Houtt.) Pak & Kawano		○							6Bb	28	29.5
	*Erigeron annuus* (L.) Pers.		○					1		6Bb	17–17.5	17.5–18
	*Erigeron bonariensis* L.		○							6Bb	16–17	17–18
	*Eupatorium makinoi* T. Kawahara & Yahara		○					1(1)		6Bb	20–21.5	20–21.5
	*Hypochaeris radicata* L.		○	(2)	(2)	(1)	(1)	1(2)	(1)	6Bb	24–25	27–28
	*Ixeridium dentatum* (Thunb.) Tzvelev subsp. *dentatum*		○							6Bb	30	33–35
	*Lactuca indica* L.		○		(1)		(2)			6Bb	33–34	36.5–38
	*Solidago altissima* L.		○	(8)	6(6)		7(7)	(1)	(1)	6Bb	21.5–23	23–24
	*Solidago virgaurea* subsp. *asiatica* (Nakai ex H. Hara) Kitam. ex H. Hara		○	(2)	1(2)	(1)	6(7)			6Bb	21.5–23	23–24
Balsaminaceae	*Impatiens noli‐tangere* L.		○	(4)	(1)	1(1)				6Bc	21–22	21–22
Brassicaceae	*Rorippa indica* (L.) Hiern		○							6Bb	24–25	24–25
Campanulaceae	*Adenophora triphylla* var. *japonica* (Regel) H. Hara		○							5Ac	36–37	37.5–41.5
Caprifoliaceae	*Abelia* × *grandiflora* (Rovelli ex André) Rehder		○	18(20)	1(3)	2(2)	(4)	3(3)	(1)	6Bb	35–40	30
Caryophyllaceae	*Stellaria neglecta* (Lej.) Weihe		○							4Ca	36–42	36–42
Commelinaceae	*Commelina communis* L.		○							2Aa	39–45	71–85
Cucurbitaceae	*Trichosanthes kirilowii* Maxim. var. *japonica* (Miq.) Kitam.		○							5Ab‐c	44–46	47–51
Fabaceae	*Albizia julibrissin* Durazz.		○							8Aa	88–92	33–34
	*Desmodium paniculatum* (L.) DC.		○							6Bb	26.5–27.5	26.5–27.5
	Fabaceae sp.			(1)						6Bb	n.d.	n.d.
	*Lespedeza thunbergii* (DC.) Nakai subsp. *thunbergii* f. *thunbergii*		○	9(20)	11(11)		(1)	(2)		6Bb	16–17	23–24
	*Lotus corniculatus* var. *corniculatus* L.		○							6Bb	16–17.5	13–14
	*Pueraria lobata* (Willd.) Ohwi		○							6Bb	26–28	26–28.5
	*Trifolium pratense* L.		○							6Bb	57–60	48–50
	*Trifolium repens* L.		○							6Bb	32–32.5	29.5–30
Gentianaceae	*Swertia japonica* (Schult.) Makino		○				1(1)			6Bb	26–27.5	28.5–30
Geraniaceae	*Geranium thunbergii* Siebold ex Lindl. & Paxton		○							6Bb	58–64	60–66
Hydrangeaceae	*Hydrangea macrophylla* f. *normalis* (E. H. Wilson) H. Hara		○							6Bb	13–15	11
Hypericaceae	*Hypericum erectum* Thunb.		○	(1)						6Bb	17–18	19.5–21
Lamiaceae	*Teucrium viscidum* var. *miquelianum* (Maxim.) H. Hara		○							6Bb	26–28	26–28.5
Liliaceae	*Lilium auratum* Lindl.		○							2Aa	85–91	100–113
Linderniaceae	*Lindernia* sp.		○							6Bb	16–17.5	16–17.5
Lythraceae	*Lagerstroemia indica* L.		○							6Bb	49	43–45
Onagraceae	*Circaea mollis* Siebold & Zucc.		○							5B	32	43–48
	*Oenothera parviflora* L.		○							5B	88–90	115–130
Orchidaceae	*Spiranthes sinensis* var. *amoena* (M. Bieb.) H. Hara		○							8B	29.5	37.5
Oxalidaceae	*Oxalis corniculata* L.		○							4Da	32–34	34–36.5
Phrymaceae	*Mazus pumilus* (Burm. f.) Steenis			(1)						6Bb	20	18
Phytolaccaceae	*Phytolacca americana* L.		○							6Bb	21	26–27.5
Pinaceae	*Pinus* spp.			(1)			(1)			3Ca	n.d.	n.d.
Plantaginaceae	*Plantago asiatica* L.		○							4Ca	21.5–24	21.5–24
	*Plantago lanceolata* L.		○							4Ca	25–27	25–27
Poaceae	*Miscanthus sinensis* Andersson		○							3Aa	35–38	35–38
Polygonaceae	*Fallopia sachalinensis* (F. Schmidt) Ronse Decr.		○							6Bb‐c	23.5–26	21–24
	*Persicaria filiformis* (Thunb.) Nakai ex W. T. Lee		○							4Da	38–41	38–41
	*Persicaria posumbu* (Buch.‐Ham. ex D. Don) H. Gross		○							4Ca	45	45
	*Persicaria* sp.		○			(1)				4Ca	41–43	41–43
	Polygonaceae sp.			(1)		(1)				4Ca	n.d.	n.d.
Primulaceae	*Lysimachia clethroides* Duby		○							6Bb	28–30	28–28.5
Ranunculaceae	*Ranunculus silerifolius* var. *glaber* (H. Boissieu) Tamura		○							4Da‐b	42–44	42–44
Rosaceae	*Agrimonia pilosa* var. *japonica* (Miq.) Nakai		○							6Bb	34–39	27–29
	*Kerria japonica* (L.) DC. f. *plena* C. K. Schneid.		○							6Bb	18–19	20–21
	Rosaceae sp.									6Bb	n.d.	n.d.
Rutaceae	*Zanthoxylum schinifolium* Siebold & Zucc.		○							6Bb	19.5–21	19.5–20
Saururaceae	*Houttuynia cordata* Thunb.		○							2Aa	16–17.5	20–21
Solanaceae	*Solanum nigrum* L.		○	(1)						6Bb	19–21	19.5–22
Vitaceae	*Causonis japonica* (Thunb.) Raf.		○							4Dd	29–33	35–38

*Note:* B = beetles; Fl = flowering present during study; L = lepidopterans; LB = large bees; MB = medium bees; n.d., no data available; OD = other dipterans; SF = syrphid flies.

^a^ Circles indicate that the plant species was in flower during the survey period. No circle indicates plant species for which only pollen was observed.

^b^ Number of captured insect individuals on the flowers of the species, with the number of insect individuals carrying pollen grains of that species in parentheses.

^c^ Type indicates the pollen classification (see Ikuse, 1956, pp. 7–16).

^d^ Size 1 is the length of the equatorial axis of the pollen (Ikuse, 1956).

^e^ Size 2 is the length of the polar axis of the pollen (Ikuse, 1956).

## APPENDIX 2 The number of observation trials performed for individuals of each functional group.

**Table:**

Functional group	Observation trial							Average ± SD^a^	Total
	4	5	6	7	8	9	10		
Large bees	8	7	3	3	3	2	1	5.85 ± 1.83	27
Medium bees	13	3	1	1			1	4.74 ± 1.52	19
Syrphid flies	3		2		1			5.33 ± 1.63	6
Other dipterans	11	3	2	4				4.95 ± 1.23	20
Lepidopterans	3	2	4		1			5.40 ± 1.26	10
Beetles	1	3	2	2			1	6.11 ± 1.76	9
Total	39	18	14	10	5	2	3	5.36 ± 1.61	91

^a^ Average number of trials ± SD required for each functional group.

## References

[aps311411-bib-0001] Alarcón, R. 2010. Congruence between visitation and pollen‐transport networks in a California plant‐pollinator community. Oikos 119(1): 35–44.

[aps311411-bib-0002] Bell, K. L. , K. S. Burgess , K. C. Okamoto , R. Aranda , and B. J. Brosi . 2016. Review and future prospects for DNA barcoding methods in forensic palynology. Forensic Science International: Genetics 21: 110–116. 26751251 10.1016/j.fsigen.2015.12.010

[aps311411-bib-0003] Brosi, B. J. , and H. M. Briggs . 2013. Single pollinator species losses reduce floral fidelity and plant reproductive function. Proceedings of the National Academy of Sciences, USA 110(32): 13044–13048. 10.1073/pnas.1307438110 PMC3740839 23878216

[aps311411-bib-0004] Chao, A. , N. J. Gotelli , T. C. Hsieh , E. L. Sande , K. H. Ma , R. K. Colwell , and A. M. Ellison . 2014. Rarefaction and extrapolation with Hill numbers: A framework for sampling and estimation in species diversity studies. Ecological Monographs 84: 45–67.

[aps311411-bib-0005] Di Pasquale, G. , M. Salignon , Y. Le Conte , L. P. Belzunces , A. Decourtye , A. Kretzschmar , S. Suchail , et al. 2013. Influence of pollen nutrition on honey bee health: Do pollen quality and diversity matter? PLoS ONE 8(8): e72016. 23940803 10.1371/journal.pone.0072016 PMC3733843

[aps311411-bib-0006] Goldblatt, P. , P. Bernhardt , and J. Manning . 1989. Notes on the pollination mechanisms of *Moraea inclinata* and *M. brevistyla* (Iridaceae). Plant Systematics and Evolution 163(3–4): 201–209.

[aps311411-bib-0007] Heinrich, B. 1975. Bee flowers: A hypothesis on flower variety and blooming times. Evolution 29(2): 325–334. 28555846 10.1111/j.1558-5646.1975.tb00212.x

[aps311411-bib-0008] Hsieh, T. C. , K. H. Ma , and A. Chao . 2020. iNEXT: Interpolation and extrapolation for species diversity. R package v. 2.0.20. Website http://chao.stat.nthu.edu.tw/wordpress/software_download/ [accessed 10 January 2021].

[aps311411-bib-0009] Huang, T. C. 1972. Pollen flora of Taiwan. National Taiwan University Press, Tapei, Taiwan.

[aps311411-bib-0010] Ikuse, M. 1956. Pollen grains of Japan. Hirokawa Press, Tokyo, Japan (in Japanese).

[aps311411-bib-0011] Inoue, T. , M. Kato , T. Kakutani , T. Suka , and T. Itino . 1990. Insect‐flower relationship in the temperate deciduous forest of Kibune, Kyoto: An overview of the flowering phenology and the seasonal pattern of insect visits. Contributions from the Biological Laboratory, Kyoto University 27(4): 377–464.

[aps311411-bib-0012] Kato, M. , T. Kakutani , T. Inoue , and T. Itino . 1990. Insect‐flower relationship in the primary beech forest of Ashu, Kyoto: An overview of the flowering phenology and the seasonal pattern of insect visits. Contributions from the Biological Laboratory, Kyoto University 27(4): 309–376.

[aps311411-bib-0013] Kearns, A. C. , and D. W. Inouye . 1993. Techniques for pollination biologists. University Press of Colorado, Boulder, Colorado, USA.

[aps311411-bib-0014] Martin, C. S. , and W. M. Farina . 2016. Honeybee floral constancy and pollination efficiency in sunflower (*Helianthus annuus*) crops for hybrid seed production. Apidologie 47(2): 161–170.

[aps311411-bib-0015] Memmott, J. , N. M. Waser , and M. V. Price . 2004. Tolerance of pollination networks to species extinctions. Proceedings of the Royal Society B: Biological Sciences 271(1557): 2605–2611. 10.1098/rspb.2004.2909 PMC1691904 15615687

[aps311411-bib-0016] Morales, C. L. , and A. Traveset . 2008. Interspecific pollen transfer: Magnitude, prevalence and consequences for plant fitness. Critical Reviews in Plant Sciences 27(4): 221–238.

[aps311411-bib-0017] Nikkeshi, A. , K. M. Hiraiwa , A. Ushimaru , K. Hoshizaki , A. Makita , and M. Inoue . 2016. Established method of deposited pollen grains on the surface of pollinated insect body. Japanese Journal of Palynology 62: 1–5 (in Japanese with English abstract).

[aps311411-bib-0018] Nikkeshi, A. , H. Inoue , T. Arai , S. Kishi , and T. Kamo . 2019. The bumblebee *Bombus ardens ardens* (Hymenoptera: Apidae) is the most important pollinator of Oriental persimmon, *Diospyros kaki* (Ericales: Ebenaceae), in Hiroshima, Japan. Applied Entomology and Zoology 54(4): 409–419.

[aps311411-bib-0019] Orford, K. A. , I. P. Vaughan , and J. Memmott . 2015. The forgotten flies: The importance of non‐syrphid Diptera as pollinators. Proceedings of the Royal Society B: Biological Sciences 282(1805): 20142934. 10.1098/rspb.2014.2934 PMC4389612 25808886

[aps311411-bib-0020] Osborne, J. L. , S. J. Clark , R. J. Morris , I. H. Williams , J. R. Riley , A. D. Smith , D. R. Reynolds , and A. S. Edwards . 1999. A landscape‐scale study of bumble bee foraging range and constancy, using harmonic radar. Journal of Applied Ecology 36(4): 519–533.

[aps311411-bib-0021] Parker, F. D. 1981. How efficient are bees in pollinating sunflowers? Journal of the Kansas Entomological Society 54(1): 61–67.

[aps311411-bib-0022] R Core Team . 2019. R: A language and environment for statistical computing. R Foundation for Statistical Computing, Vienna, Austria. Website https://www.R‐project.org/ [accessed 30 December 2020].

[aps311411-bib-0023] Sakai, S. , K. Momose , T. Yumoto , T. Nagamitsu , H. Nagamasu , A. A. Hamid , and T. Nakashizuka . 1999. Plant reproductive phenology over four years including an episode of general flowering in a lowland dipterocarp forest, Sarawak, Malaysia. American Journal of Botany 86(10): 1414–1436. 10523283

[aps311411-bib-0024] Tur, C. , J. M. Olesen , and A. Traveset . 2015. Increasing modularity when downscaling networks from species to individuals. Oikos 124(5): 581–592.

[aps311411-bib-0025] Yamazaki, K. , and M. Kato . 2003. Flowering phenology and anthophilous insect community in a grassland ecosystem at Mt. Yufu, Western Japan. Contributions from the Biological Laboratory, Kyoto University 29(3): 255–318.

